# Formative Assessment Review Project

**DOI:** 10.1192/bjo.2025.10264

**Published:** 2025-06-20

**Authors:** Cherie Collins

**Affiliations:** RCPsych, London, United Kingdom

## Abstract

**Aims:** Following the approval of the Assessment Strategy Review (ASR) in February 2023, the Formative Assessment Working Group (FAWG) was created to discuss areas for consideration, in relation to formative assessment.

The aim was to consider the broad range of the College’s assessments, including written examinations, the Clinical Assessment of Skills and Competencies (CASC) and formative assessments undertaken throughout psychiatric training in the workplace. Whilst the ASR had a broad scope, one of the recommendations was to review formative assessment in detail, due to the varied nature of specialty and sub-specialty specific assessments and the differing experiences of resident doctors in the workplace.

**Methods:** The Formative Assessment Working Group (FAWG) met throughout 2023 and identified four specific areas for further consideration, development and implementation:

The introduction of enstrustability scales; a behaviourally anchored ordinal scale based on progression to competence, as part of workplace-based assessments (WPBAs).

Embedding formulation skills throughout training.

The introduction of feedback from patient and carers for resident doctors.

Consideration of guided supervision sessions relating to caseload-based discussion.

**Results:** Entrustability scales as part of WBPAs: An entrustability scale (ES) would not be suitable to all assessment types. Therefore, an adaptation of ES will be introduced to CS and ES reports. WPBA will see an improved version of the current Likert scale. Embedding formulation skills throughout training: Formulation training will be incorporated into case presentations. Resident doctors will be advised to undertake this yearly to demonstrate progression. For ST4+ trainees, one case-based discussion (CBD) will be replaced with a case presentation (CP), with the provision of presenting to MDTs. Introduce feedback from patients and carers for resident doctors: Resident doctors will be expected to collect one set of Multisource feedback (MSF) responses during core training and at least once per speciality training year. This will apply across all psychiatric specialties, including those who undertake dual training. The feedback should also form part of ARCP considerations. Introduce guided supervision session relating to caseload-based discussion: Direct Observation of Non-Clinical Skills (DONCS) will now feature HLOs 1–9 as part of the ‘skills observed’ part of the online portfolio. This will allow for resident doctors to capture skills and experience that aren’t covered elsewhere in their portfolios.

**Conclusion:** The changes should allow resident doctors to demonstrate the skills and knowledge acquired across their training. They will also be able to demonstrate a clear understanding of their progression and benefit from a variety of feedback opportunities.

